# *Lacticaseibacillus casei* T1 attenuates *Helicobacter pylori-*induced inflammation and gut microbiota disorders in mice

**DOI:** 10.1186/s12866-023-02782-4

**Published:** 2023-02-11

**Authors:** Zhihao Yu, Mei Cao, Jingshan Peng, Daoyan Wu, Shu Li, Chengmeng Wu, Liting Qing, Andong Zhang, Wenjie Wang, Min Huang, Jian Zhao

**Affiliations:** 1 Key Laboratory of Biological Resource and Ecological Environment of Chinese Education Ministry, College of Life Sciences, Sichuan University, No.24 South Section 1, Yihuan Road, Chengdu, 610064 People’s Republic of China; 2 Core Laboratory, School of Medicine, Sichuan Provincial People’s Hospital Affiliated to University of Electronic Science and Technology of China, Chengdu, 610072 People’s Republic of China; 3 Department of Microbiology, School of Basic Medical Sciences, Guizhou Medical University, Guiyang, 550025 People’s Republic of China; 4 Irradiation Preservation Technology Key Laboratory of Sichuan Province, Sichuan Institute of Atomic Energy, Chengdu, 610101 People’s Republic of China

**Keywords:** *H. pylori*, *Lacticaseibacillus casei*, Probiotics, Inflammation, Oxidative stress

## Abstract

Probiotics are defined as live microbial food elements that are beneficial to human health. *Lacticaseibacillus casei* T1 was considered to have potential as a bioactive ingredient in functional foods, which was isolated from kurut. Previous research by our group proved that *L. casei* T1 could prevent inflammatory responses caused by *Helicobacter pylori*. This study aimed to investigate whether treatment with *L. casei* T1 resulted in a suppressive effect on *H. pylori*-induced oxidative stress and inflammatory responses. The results showed that treatment with *L. casei* T1 could relieve *H. pylori*-induced overexpression of inflammatory cytokines in GES-1 cells. Experiments in animals suggested that taking long-term *L. casei* T1 could reduce oxidative stress and inflammatory cytokines and improve *H. pylori-*induced gastric mucosal damage. Furthermore, taking *L. casei* T1 could increase the relative abundance of beneficial intestinal bacterium (*Lachnospiraceae* and *Odoribacter*) of *H. pylori*-infected mice and help in maintaining the balance of intestinal microflora.

Collectively, *L. casei* T1 had certain degrees of therapeutic effect against *H. pylori*. In the future, it combined with antibiotics for *H. pylori* eradication deserves further study.

## Introduction

*Helicobacter pylori,* a strain of microaerobic bacteria, is a curved spiral-shaped bacterium isolated from the stomach [1]. More than half of the world population lives with *H. pylori*, with an infection rate of about 25% in developed countries and 80% in developing countries [2]. And *H. pylori* is recognized as the most common cause of chronic gastritis, gastric ulcer, duodenal ulcer and is also an important pathogenic factor in gastric cancer [3, 4]. It is considered a class I human carcinogen [5, 6]. A triple or quadruple regimen is a routine treatment for *H. pylori* infection [7–9]. Along with extensive use of antibiotics in clinical and agricultural settings over the past few decades, the ever-increasing number of drug-resistant *H. pylori* and lack of alternative antibiotics lead to increasing difficulties in eradication therapy. Reinfection rates are also high in developing countries [10]. Moreover, misuse and overuse of antibiotics not only led to many adverse consequences, for instance diarrhea, nausea and drug eruption, but also resulted in an imbalance in the gastrointestinal microbiota [2, 11]. Therefore, it is necessary to develop new complementary therapeutic strategies. Currently, probiotics are extensively studied for their beneficial effects in prevention and treatment of many prevailing diseases [12]. Probiotics have antimicrobial activity, produce bacteriocins, have antitoxin effects, enhance the intestinal barrier function and exercise immune modulation [13]. Because of the multiple bactericidal mechanisms of probiotics, taking them does not cause bacterial resistance. Thus, probiotic adjuvant therapy may become a new choice in *H. pylori* infection [14–16].

Probiotics can colonize the gastrointestinal tract and beneficially affect the host by balancing the gastrointestinal flora, reducing inflammation and improving immune function [17]. In contrast to the currently used antibiotics, bacteriocins generated by lactic acid bacteria (LAB) are often considered natural and safe, because they have been naturally present in fermented foods [18]. LAB is also used as fermenting agent, and they are extensively used in the manufacture of fermented milk products, wine and pickled vegetables [19, 20]. The safety of these microorganisms has not been questioned, and reports of these bacteria being harmful are very rare [18]. Studies in vitro have documented two strains of *Limosilactobacillus reuteri* isolated from the human body have good viability under gastrointestinal conditions, as well as strong resistance to *H. pylori* and antioxidant activity [21]. Studies showed that *H. pylori* infection was effectively treated and controlled, when a fermented milk-based product which contains *Bifidobacterium lactis* Bb12 and *Lactobacillus acidophilus* Bb12 was consumed for six weeks [21]. Some research verified ingestion of probiotics alone could reduce colonization of *H. pylori*, but it couldn't be completely excluded from the stomach. The eradication rate of *H. pylori* could be enhanced by 10% when probiotics combined with antibiotic [22–24]. In addition, taking probiotics can reduce the side effects of antibiotic treatment [25]. It is well accepted that long-term consumption of food containing probiotics is beneficial to human health.

*Lacticaseibacillus casei* T1 is a probiotic strain isolated from kurut described from Qinghai-Tibet plateau, which has excellent acid tolerance and a high survival rate in the stomach [26, 27]. The acid generating capacity of *L. casei* T1 is strong and can reduce the pH of the fermentation broth to well below 4.0 after 48 h incubation. It also can produce an extracellular bacteriocin that possesses the activities against drug-resistant bacteria, which has high thermal and pH stability [26]. Our previous studies have confirmed that taking *L. casei* T1 in advance can contribute to ameliorates inflammatory response caused by *H. pylori *in vivo [28]. Nevertheless, the difference between prevention and treatment might be that the latter provides a more direct and visible effect. In the present study, we investigated the therapeutic effect of *L. casei* T1 after *H. pylori* infection in vivo.

## Materials and methods

### ***Bacterial strains and their cultivation***

All the strains were derived from Key Laboratory of Biological Resource and Ecological Environment of Chinese Education Ministry. *L. casei* T1 was cultured on MRS agar solid medium or MRS liquid medium for 24 h at 37℃. ​The strain of *H. pylori* used in this paper is a clinical strain isolated from a patient with gastric ulceration and moderate gastritis at the People's Hospital of Sichuan Province.

*H. pylori* and the remaining strains were cultured according to the method reported by Wu et al. [28] and Luo et al. [26]. *Escherichia coli* (China Center of Industrial Culture Collection; CICC 10,354) and *Staphylococcus aureus* (CICC 10,201) were isolated from spoiled milk. *Escherichia coli*, *Salmonella typhimurium*, *Staphylococcus aureus*, *Bacillus subtilis* and *Bacillus cereus* were propagated in LB broth (Qingdao Haibo Biotechnology *Co., Ltd*, HB0128) at 37℃ for 24 h. MRS Broth medium (Qingdao Haibo Biotechnology *Co., Ltd,* HB0384-1) was used to culture *Lactobacillus bulgaricus*, *Lactobacillus acidophilus*, and *Levilactobacillus brevis* for 24 h at 37℃.

### ***Bacteriostatic spectrum detection***

*L. casei* T1 was cultured for 48 h at 37℃ in MRS liquid medium and the supernatant were collected by centrifugation at 8,000 g for 10 min. Then it was filtered with a 0.22um filter to remove residual organisms. Then it was tested for antimicrobial activities by the well-diffusion assay [29]. The melted MRS agar medium was first poured onto the plate and solidified as a bottom layer. Then, 1 mL of the bacterial solution (1 × 10^7^ cfu/mL) was added, and LB agar medium was added at about 50℃. Shake the plate gently to mix and set horizontally until set. Holes were punched by Sterile hole punchers (d = 7 mm) and 100 µL *L. casei* T1 fermentation supernatant was added to each hole and cultured at 37℃ for 24 h. Bacteriostatic activity was determined by measuring the diameter of inhibitory zones.

### ***Cell culture***

GES-1 cells are used as a reliable in vitro model of human gastric epithelium. The source and culture conditions of GES-1 cells refer to the report of Wu et al. [28]. GES-1 cells (5 × 10^5^ cells/well) were seeded in 6-well plates and cultured 24 h. Then, the GES-1 cells were co-cultured with *H. pylori* (5 × 10^7^ Colony Forming Unit, CFU/well) or *L. casei* T1 (5 × 10^7^ CFU/well) for 24 h. Afterwards, the GES-1 cells co-cultured with *H. pylori* were treated with *L. casei* T1 (5 × 10^7^ CFU/well) or amoxicillin (5 mg/L) for 1 day. The gathered cells and supernatants were used for qPCR and Enzyme Linked Immunosorbent Assay (ELISA).

### Animal experiments

Eight-ten weeks old female Balb/c mice were purchased from Chengdu Dashuo Experimental Animal Co., Ltd. (Chengdu, China). Mice were housed in standard conditions with food and water ad libitum in the conventional vivarium. After adaptive feeding for 7 days, 30 mice were randomized into 2 groups receiving the first pretreatment as described below: ① Group C(8 mice): 500 μL normal saline were orally perfused to mice once every other day for 4 weeks; ② Group H(22 mice): mice were administered 500 µl *H. pylori* suspension (2 × 10^9^ cfu/mL) by intragastric gavage alternate day for 4 weeks. After the first pretreatment, 6 randomly selected mice in Group H were fasted overnight for gastric tissue sampling. Prior literature suggested DNA of *H. pylori* could be discovered in infected antrum stomach tissues [30]. In order to evaluate the status of *H. pylori* infection, gastric tissues were used for routine rapid urease testing and polymerase chain reaction (PCR). DNA was extracted by TIANamp Bacteria DNA Kit purchased from Tiangen Biotech Co., Ltd. The extracted DNA was subject to PCR using specific primers of the *H. pylori* *vacA* gene. Primers were F-*vacA*: GCCGATATGCAAATGAGCCGC, R-*vacA*: CAATCGTGTGGGTTCTGGAGC. The products were identified by 1% agarose gel electrophoresis and anticipated being 678 base pairs.

When the *H. pylori* infection model was successfully established, 8 mice were arbitrarily selected from Group H and assigned to Group H + T1. Meanwhile, the mice underwent the second treatment as described below: ① Group C(8 mice) and Group H(8 mice): 500 μL normal saline were orally perfused once daily for 2 weeks; ② Group H + T1(8 mice): 500 μL bacterial suspension of *L. casei T1* (2 × 10^9^ cfu/mL) was administered by oral gavages once a day for 2 weeks. Fecal samples were collected by placing the mice individually in aseptic cages on the last day of the gavage. Mice were euthanized after 12 h of fasting at the end point of the experiment to collect blood, and tissues. 100 ~ 200 μL of blood was collected in anticoagulant tubes (Ethylene Diamine Tetraacetic Acid, EDTA) for blood routine examinations. The rest of blood collected in anticoagulant-free tubes was allowed to clot overnight at 4℃. And serum was prepared by centrifugation at 3000 g at 4℃ for 20 min, stored at − 80℃. The gastric tissue was collected for qPCR, the rapid urease test, Hematoxylin and eosin (HE) staining and immunohistochemical (IHC) experiments.

### ***Analysis of gene expression***

Total RNA was extracted by an RNA extraction kit (Thermo Fisher Scientific, PureLink™ RNA, 12183018A). RNA was reverse transcribed by Reverse Transcription kit (TaKaRa, PrimeScript RT reagent Kit with gDNA Eraser, RR047A). SYBR Green real time PCR was performed using QuantStudio3. Reactions were run at 50℃ for 2 min; 95℃for 30 s; then 40 cycles at 95℃ for 15 s, 60℃ for 30 s and 72℃ for 10 s. The GAPDH gene was used as an internal reference gene. Primers of selecting genes for qPCR designed through Oligo7. The primers of GES-1 were as follows: F-*GAPDH* TGCACCACCAACTGCTTAGC, R-*GAPDH* GGCATGGACTGTGGTCATGAG, F- tumor necrosis factor (*TNF-α*) GAGGCCAAGCCCTGGTATG, R-*TNF-α* CGGGCCGATTGATCTCAGC, F- interleukin 6 (*IL6*) CTTCGGTCCAGTTGCCTTCT, R-*IL6* TGGAATCTTCTCCTGGGGGT, F-*IL8* ACACTGCGCCAACACAGAAA, R-*IL8* CAACCCTCTGCACCCAGTTT. The primers of mice were as follows: F-*GAPDH* ATG-GTGAAGGTCGGTGTGAAC, R-*GAPDH*GGAGTCATACTGGAACAT-GTAGACC, F-*TNF-α* CCTGTAGCCCACGTCGTAG, R-*TNF-α* GGGAGTAGACAAGGTACAACC-C, F-*MUC5AC* CTGTGACATTATCCCATAAGCCC, R-*MUC5AC* AAGGGGTATAGCTGGCCTGA.

### Analysis of protein expression

The cell supernatants were detected by Human IL6 ELISA KIT, Human TNF-α ELISA KIT, Human IL8 ELISA KIT and Human C–C motif chemokine ligand 2 (CCL2) ELISA KIT purchased from Chengdu Pengshida Experimental Supplies Co., Ltd. The stored serum samples were thawed on ice. Subsequently, the concentrations of CRP, MDA, GSH-PX and SOD in serum were quantified by MOUSE ELISA kits from the same company.

### Routine blood examination

As mentioned earlier, the blood samples just collected in anticoagulant tubes (EDTA) was sent to Harmony Yijia Pet Hospital (chengdu, China) within two hours, and the routine blood examination was performed by Celltac Alpha MEK-6400 series hematology analyzers (Nihon Kohden, MEK-6400).

#### HE staining and IHC experiments

HE staining and IHC staining experiments were performed according to standard histological protocols. Anti NF-κB/p65 was purchased from Wanleibio Co., Ltd. Goat anti-rabbit secondary antibody was purchased from Beyotime Biotechnology Co., Ltd.

### Gut microbiota analysis

The collected fecal samples were shipped to Beijing Baimaike Biotechnology Co., Ltd on dry ice for *16S* ribosomal DNA sequencing and analyses. *338F* and *806R* were used to amplify the V3-V4 region of the *16S* rRNA gene.

### Statistics

Data are expressed as means ± SD. Prism 8.0.2 software was used for statistics analysis. To determine the statistical significance, *P* values were determined by ANOVA or Student’s t-test. *P* < 0.05 was considered as significant.

## Results

### The antibacterial spectrum of fermentation broth

The effect of the bacteriostatic experiment is shown in Table 1. The fermentation liquor of *L. casei* T1 exhibited comparatively good antibacterial activity against resistant strains of *S. aureus* and *E. coli.* Further analysis showed that *L. casei* T1 had antagonistic effects not only on lactic acid bacteria with a high degree of relatedness*,* but also on gram-positive bacteria and gram-negative bacteria. Excessive proliferation of harmful bacterium reduces food shelf life and increases foodborne illness [31]. Microbial contamination will bring health problems and huge economic losses [32]. Nevertheless, the public demands to reduce the use of chemical preservatives or additives in food, increasing attention is being paid to the natural additives [33, 34]. So the antimicrobial activity of *L. casei* T1 indicated it could be used in agriculture and food applications. The fermentation liquor of *L. casei* T1 was also active in repressing *H. pylori* growth. The above results showed broad spectrum antimicrobial activity of *L. casei* T1*’s* fermentation broth.

**Table 1:** Antibacterial spectrum of *L. casei* T1’s fermentation broth

Indicator bacteria	Bacteriostatic activity	Indicator bacteria	Bacteriostatic activity
*H. pylori*	+ +	*B. cereus*	+ +
*Escherichia coli*	+ +	*L. bulgaricus*	+ +
*S. typhimurium*	+ +	*L. acidophilus*	+ +
*S. aureus*	+ + +	*L. brevis*	+
*B. subtilis*	+ + +		

‘ + , +  + , +  +  + ’ represents the size of the bacteriostatic circle diameter. ‘ +  +  + ’: the diameter is over 15 mm, ‘ +  + ’: the diameter is 10-15 mm, ‘ + ’: the diameter is 8-10 mm

### *L. casei* T1 inhibited the overexpression of inflammatory cytokines by *H. pylori* infection

**Fig. 1:**
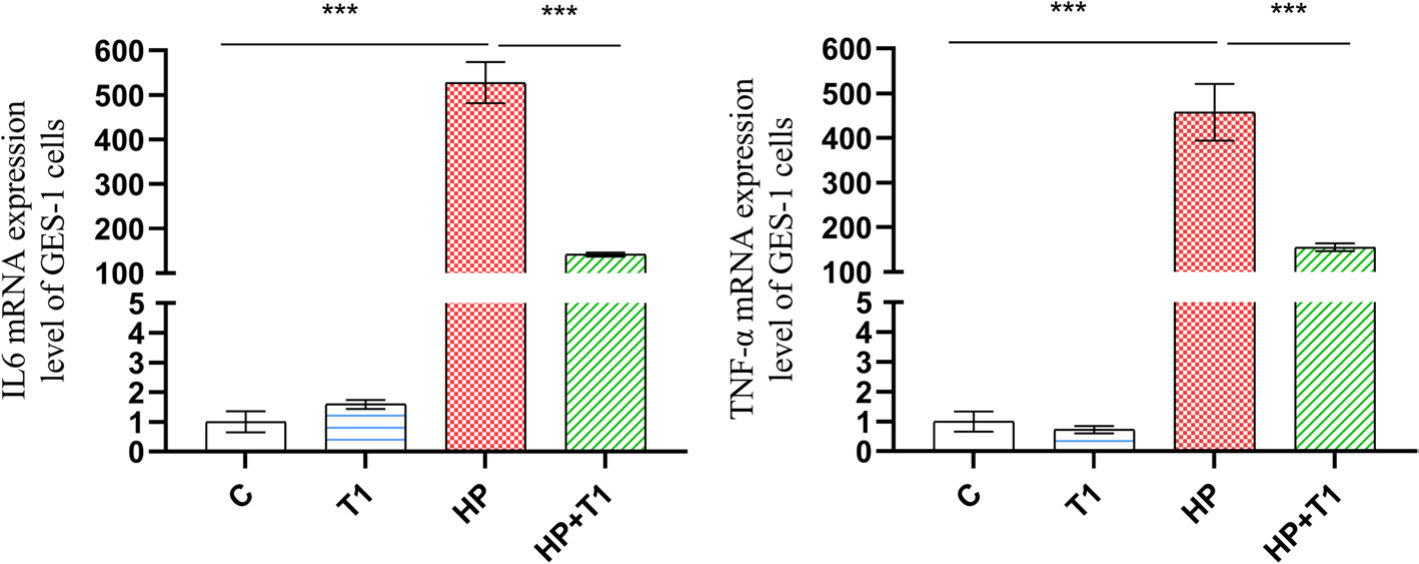
Impact of *L. casei* T1 on inflammatory cytokines gene expression in vitro*. The r*esults are the means and SDs (*N* = 3) and *P* values were calculated by *t* tests. *P* values: * *P* < 0.05; ** P < 0.01; *** *P* < 0.001. C: blank control; T1: treated with *L. casei* T1 for 48 h; HP: treated with *H. pylori* for 48 h; HP + T1: treated with *H. pylori* for 24 h and *L. casei* T1 for another 24 h

**Fig. 2:**
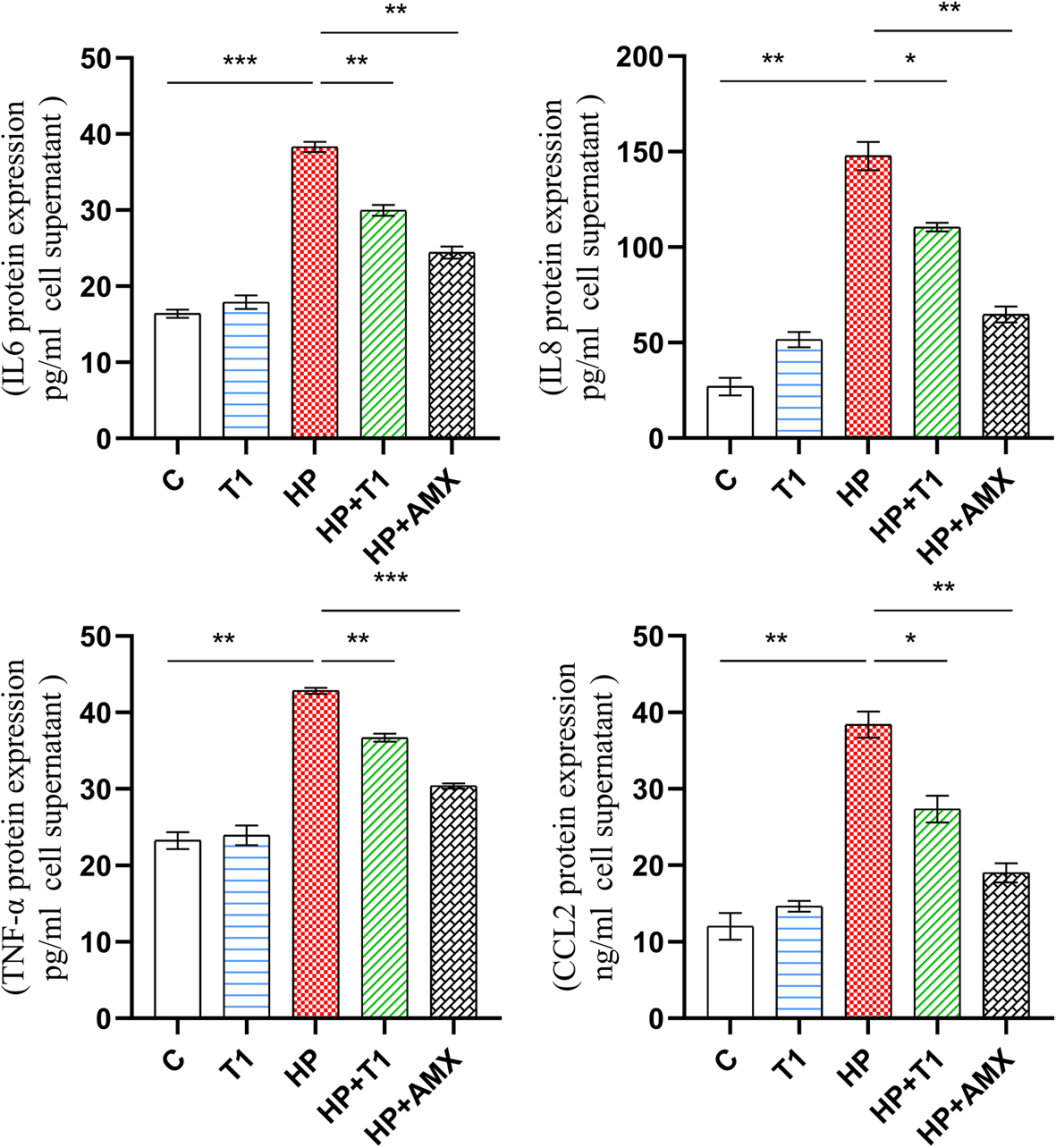
Impact of *L. casei* T1 on inflammatory protein in vitro*. The r*esults are the means and SDs (*N* = 3) and* P* values were calculated by *t* tests. *P* values: * P < 0.05; ** *P* < 0.01; *** *P* < 0.001. C: blank control; T1: treated with *L. casei* T1 for 48 h; HP: treated with *H. pylori* for 48 h; HP + T1: treated with *H. pylori* for 24 h and *L. casei* T1 for another 24 h; HP + AMX: treated with *H. pylori* for 24 h and amoxicillin (5 mg/L) for another 24 h

By far, many reports have shown that *H. pylori* infection induces high expression of inflammatory factors such as IL6 and TNF-α [35, 36]. By Fig. 1, it was clearly seen the *IL6* and *TNF-α* mRNA expression levels of Group HP + T1 were markedly reduced compared to Group H. CCL2, also known as MCP-1, was found to possess mighty chemotactic ability to recruit monocytes and macrophages [37]. It also had a significant influence in many acute and chronic inflammatory diseases [38, 39]. Studies showed that CCL2 could activate monocytes to unleash inflammatory cytokines and provoke respiratory burst that can lead to generate excess reactive oxygen species (ROS) and harm cells [40]. As presented in Fig. 2, the overexpression of inflammatory proteins IL6, IL-8, TNF-α and CCL2 were inhibited by *L. casei* T1 and amoxicillin (5 mg/L) treatment. These findings clearly indicated that *L. casei* T1 could attenuate the inflammatory response induced by *H. pylori *in vitro*.*

### H. pylori infection model

**Fig. 3:**
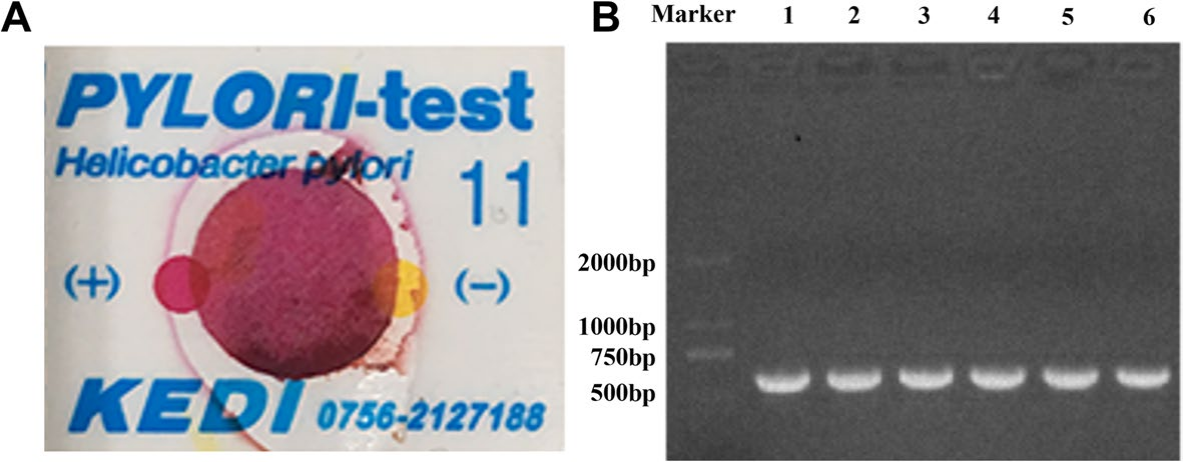
Detection of *H. pylori* infection model. **A**: Results of rapid urease test (turn red within a minute means positive, turn red within three minutes means light positive, yellow means negative); **B**: Electrophoresis of PCR-amplified products (*vacA*)

Following the completion of the first pretreatment of mice, assessment of *H. pylori* colonization was identified by rapid urease test and PCR. As indicated in Fig. 3 (A), the dipsticks appeared red within a minute. After extracting the DNA of sampled gastric mucosae, PCR was performed with specific primers of *H. pylori*'s *vacA* gene. After the reaction, the product was consistent with the expectation at 678 bp as indicated in Fig. 3 (B). Overall, we concluded that Balb/c mice of Group H were successfully infected with *H. pylori.*

### Rapid urease test

**Fig. 4:**
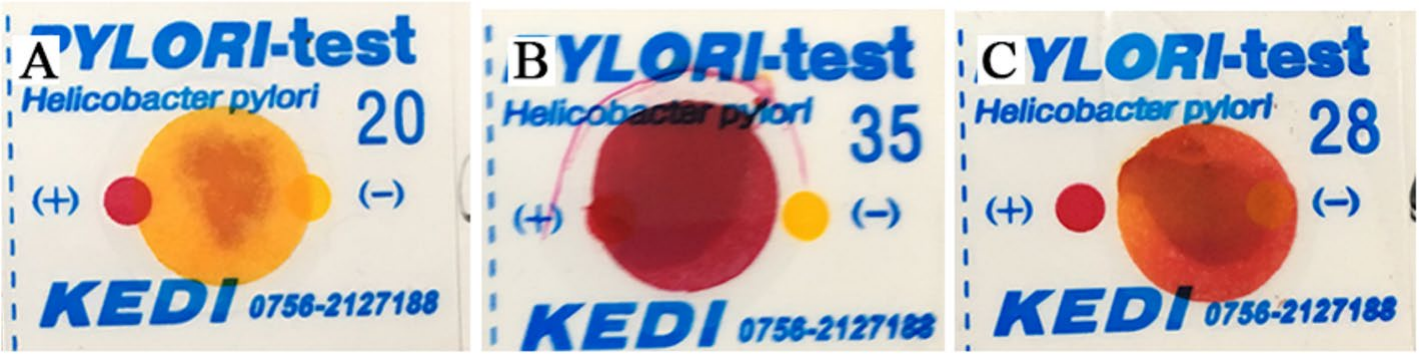
Results of rapid urease test in mouse gastric tissues. Turn red within a minute means positive, turn red within three minutes means light positive, yellow means negative. **A**: Group C; **B**: Group H; **C**: Group H + T1

After the second treatment, all mice were euthanized and the gastric tissues were evaluated by the rapid urease test. It can be seen from Fig. 4 that gastric samples of Group C presented yellow color and Group H appeared red color. The color of Group H + T1 lies between the two, which means the severity of *H. pylori* infection of Group H + T1 is less serious than that of Group H.

### Routine blood tests

The results of the routine blood tests were given in Table 2. By comparing the experimental date, the numbers of white blood cells (WBC) and neutrophils (NEU) of Group H and Group H + T1 were substantially higher than Group C (*P < ​ 0.05, # *P* < ​ 0.05). However, there were no noticeable differences between Group H and Group H + T1. Overall, these results indicated that there were obvious inflammatory reactions in Group H and Group H + T1.

**Table 2:** The number of white blood cell (WBC) and neutrophils (NEU)

Numbers(10^9^/L)	Group C	Group H	Group H + T1
WBC	4.75 ± 0.42	10.07 ± 0.88*	9.73 ± 0.50*
NEU	0.35 ± 0.05	0.98 + 0.08^#^	0.95 ± 0.08^#^

The results are the means and SDs (*N* = 3 ~ 6) and* P* values were calculated by one-way ANOVA

* *P* < 0.05, *vs*. Group C

# *P* < 0.05, *vs*. Group C

### ***L. casei ***T1*** alleviated H. pylori-induced gastric mucosal inflammation***

**Fig. 5:**
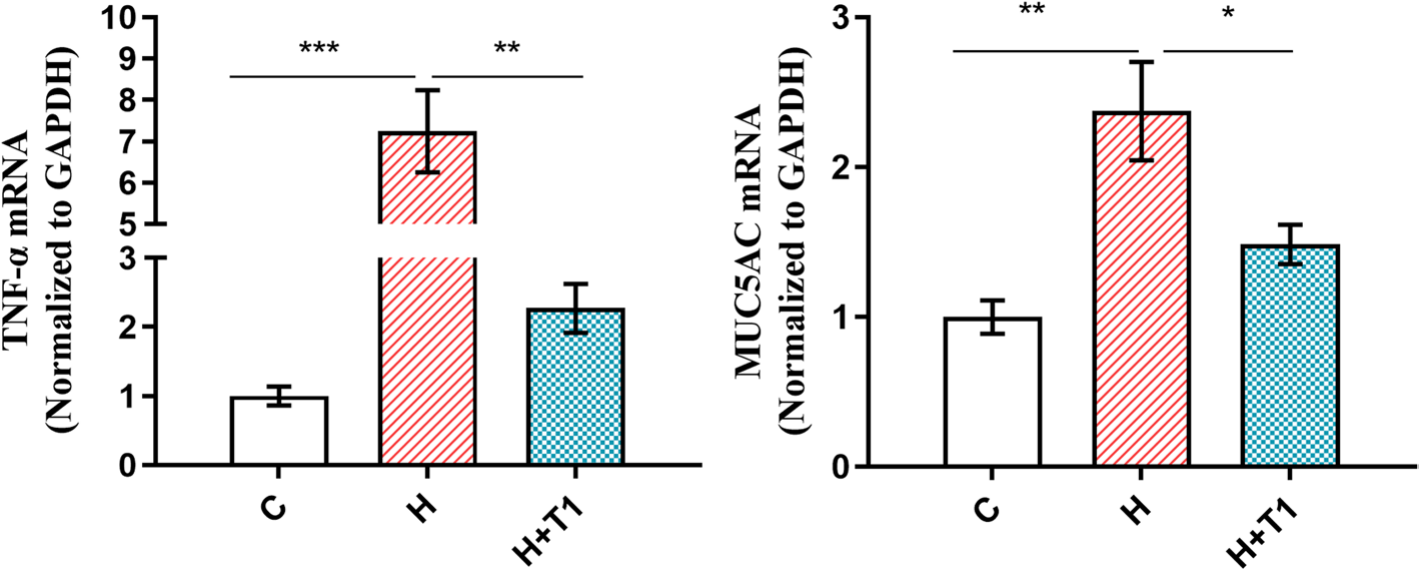
The *TNF-α* and *MUC5AC* mRNA expression in mice. The results are the means and SDs (*N* = 3 ~ 6) and *P* values were calculated by *t* tests. *P* values: * *P* < 0.05; ** *P* < 0.01; *** *P* < 0.001. C: Group C; H: Group H; H + T1: Group H + T1

**Fig. 6:**
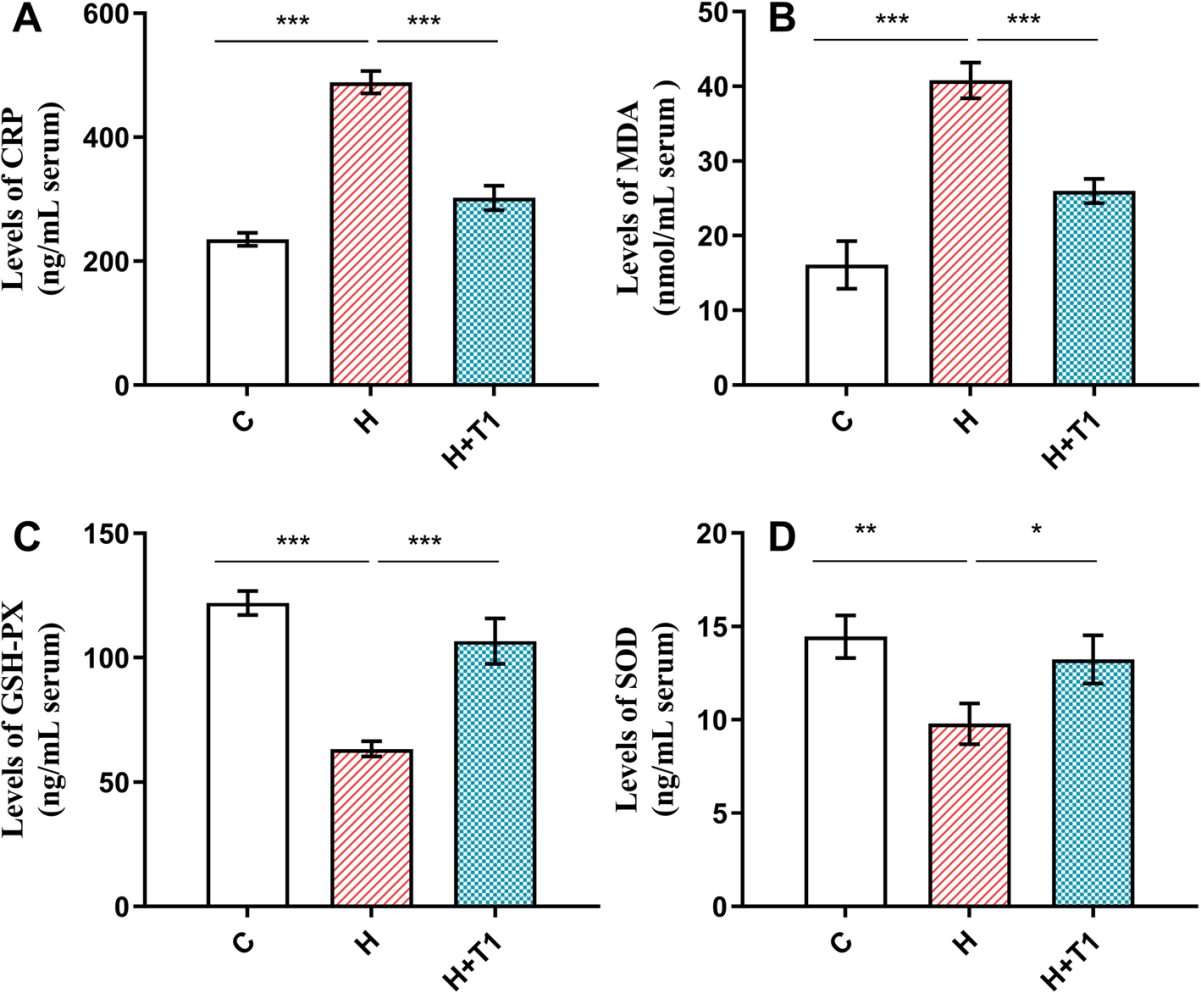
Results of ELISA detection of mouse serum. The results are the means and SDs (*N* = 3 ~ 6) and *P* values were calculated using *t* tests. P values: * P < 0.05; ** *P* < 0.01; *** *P* < 0.001. C: Group C; H: Group H; H + T1: Group H + T1

Previous studies have indicated that mucin 5AC (MUC5AC) played a role in the adhesion of* H pylori* to the gastric mucosa [41, 42]. It was recently shown that MUC5AC was appreciably elevated in response to live *H. pylori* and had a positive association with *H. pylori* adhesion [43]. It can be seen from Fig. 5, Compared with Group H, the mRNA expression of *TNF- α* and *MUC5AC* in Group H + T1 decreased significantly. (P < 0.05). C-reactive protein (CRP) whose production is stimulated by IL6 is an acute reactive protein in humans and increases rapidly in the presence of inflammation [44–46]. As shown in Fig. 6 (A), the content of CRP in the serum of Groups C, H, and H + T1 was 235.40, 488.75 and 302.00 ng/mL, respectively. Compared to Group C, the CRP concentration of Group H and Group H + T1 was dramatically increased (P < 0.05). And the CRP concentration of Group H was obviously higher than Group H + T1 (P < 0.05). This could be illustrated as follows: *L. casei* T1 could effectively restrain *H. pylori* colonization in gastric mucosa and improve inflammation caused by *H. pylori*.

Long-term inflammation process increases ROS production, causing oxidative stress the severity can be reflected by malondialdehyde (MDA) [47, 48]. As can be seen from Fig. 6 (B), the MDA of Group H + T1 was clearly lower than Group H (P < 0.05). Antioxidant enzymes are commonly used to scavenge ROS, such as glutathione peroxidase (GSH-PX) and superoxide dismutase (SOD) [49]. GSH-PX plays a crucial role in detoxifying H_2_O_2_, a major type of ROS [50]. SOD efficiently detoxifies superoxide in the cytoplasm and mitochondria and is a major cellular defense against ROS [51]. According to Fig. 6 (C and D), *L. casei* T1 could effectively improve the low expression of GSH-PX and SOD induced by *H. pylori* infection. Thus, the outcomes confirmed that *L. casei* T1 can alleviate oxidative stress.

**Fig. 7:**
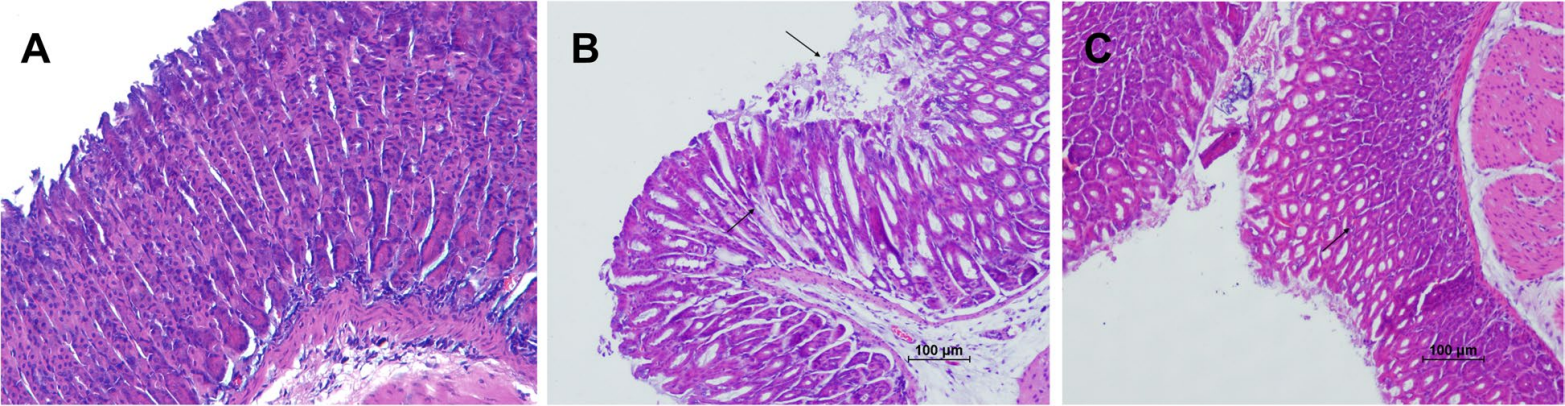
The results of gastric tissue HE staining. **A**: Group C; **B**: Group H; **C**: Group H + T1

**Fig. 8:**
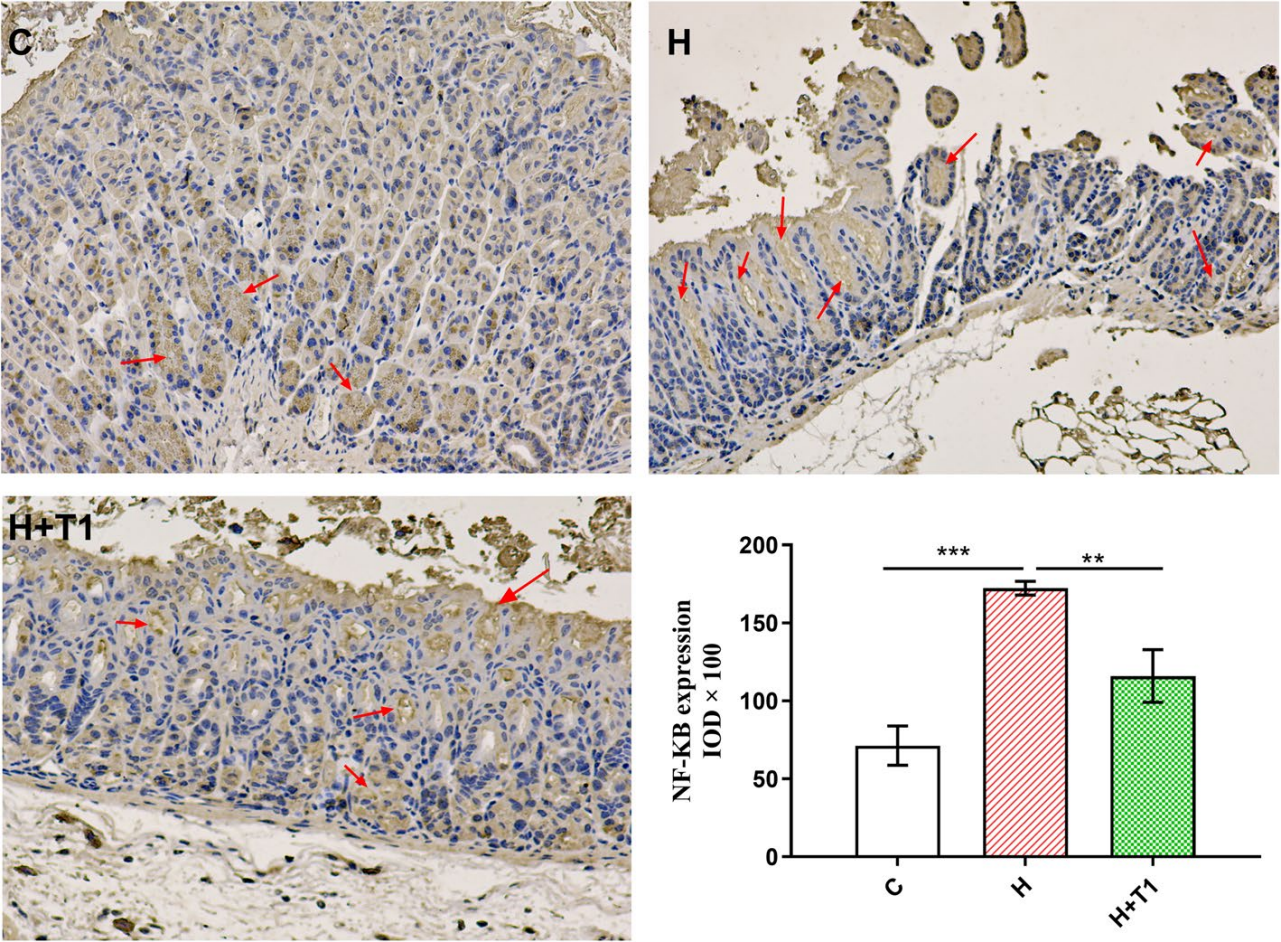
The NF-κB protein expression. The results are the means and SDs (*N* = 3 ~ 6) and* P* values were calculated by *t* tests. *P* values: * P < 0.05; ** *P* < 0.01; *** P < 0.001.C: Group C; H: Group H; H + T1: Group H + T1

*H. pylori* causes widespread gastric disease via directly injuring the gastric mucosa [30, 52]. HE staining results are shown in Fig. 7. The gastric mucosa of Group C was normal morphology and had no mucosal shedding and erosion (Fig. 7A). The result of Group H shows red blood cells can be seen in the gastric mucosa and there are cell necrosis and falling off the surface of the mucosa (Fig. 7B). Meanwhile, the gastric mucosa of Group H + T1 is visible with red blood cells and a slight amount of shedding cells (Fig. 7C). It follows that continuously feeding *L. casei* T1 alleviated mild gastric mucosal injury. NF-κB is seen to be a prototypical proinflammatory signaling pathway, greatly based on the activation of NF-kB by proinflammatory cytokines, for instance, TNF-a and IL-1 [53]. We could see from Fig. 8 that the NF-κB protein expression of Group H was 2.42 times of Group C, indicating *H. pylori* infection generated gastritis in mice. And, the NF-κB protein expression of Group H + T1 was as 0.67 times as Group H (*P* < 0.01), implying *L. casei* T1 had the capacity to directly resist *H. pylori* infection.

### Effects of L. casei T1 on gut microbiota in mice

**Fig. 9:**
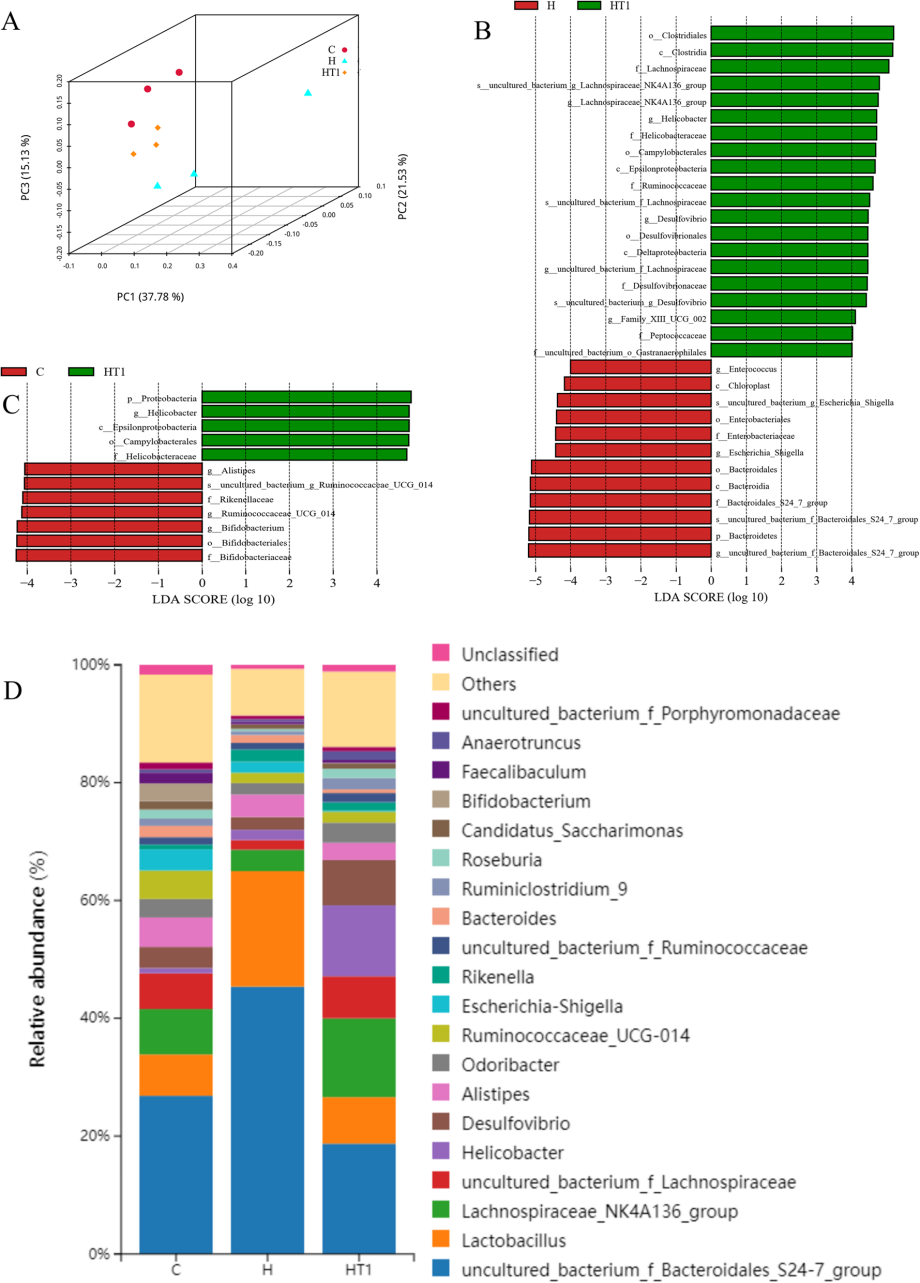
Effects of *L. casei* T1 treatment on gut microbiota of mice. **A** Principal coordinates analyses (PCoA) provide a visual illustration of grouped samples; **B**: Linear discriminant analysis (LDA) effect size (LEfSe) identified taxa most characteristic in H and HT1 groups; **C**: Linear discriminant analysis (LDA) effect size (LEfSe) identified taxa most characteristic in C and HT1 groups; **D**: Relative abundances of gut flora at genus level. C: Group C; H: Group H; HT1: Group H + T1

To identify microbial genera associated with *L. casei* T1 treatment, mouse fecal microbial composition was examined by 16S rRNA sequence. As can be seen from Fig. 9 (A), the 3D plot of PCoA provided a visual illustration of grouped samples. PC1 explained 37.78% of the variation, PC2 explained 21.53% and PC3 explained 15.13%. It demonstrated relatively significant differences in gut flora between groups. The differences of the intestinal microbial communities of groups at genus level was shown in Fig. 9 (D). Compared with Group C, the relative abundance of *Bacteroidales_*S24-7, *Lactobacillus* and *Helicobacter* of Group H increased, and the relative abundance of *Lachnospiraceae_*NK4A136*, Lachnospiraceae, Desulfovibrio* and *Alistipes* reduced in genus level. Compared to Group H, the relative abundance of *Bacteroidales_*S24-7*, Lactobacillus* and *Alistipes* of Group H + T1 reduced, and the relative abundance of *Lachnospiraceae_*NK4A13*6, Lachnospiraceae, Helicobacter* and *Odoribacter* increased in genus level. The results of the significant differences analysis between groups were shown in Fig. 9 (B and C). Combined with relative species abundance, the main differential strains between the Group H and Group H + T1 were *Bacteroides, Escherichia_Shigella, Enterobacter* and *Helicobacter*. The main differential strains between Group C and Group H + T1 were *Bifidobacterium* and *Helicobacter*.

*Bacteroides* represent opportunistic pathogens in infectious diseases [54]. *Escherichia_Shigella* and *Enterobacter* are recognized in mammals as being both potentially pathogenic gut commensals [55]. After *H. pylori* infection in mice, the relative abundance of *Bacteroides, Escherichia_Shigella* and *Enterobacter* in the gut decreased. This showed that *H. pylori* could inhibit the reproduction of opportunistic pathogens. And treatment of *H. pylori* infection with *L. casei* T1 further suppressed the relative abundance of *Bacteroides, Escherichia_Shigella* and *Enterobacter.* Studies have identified *Lachnospiraceae* as potential promoters of gut health; for instance, it has been shown to have protective effects against *Clostridioides difficile* infection in preclinical studies [56]. Moreover, many *Lachnospiraceae* produce butyrate, which is a nutrient for the gut [57]. *Odoribacter* is generally considered to have a beneficial effect against inflammation [58]. In addition, decreased *Odoribacter* levels were found in insulin-resistant people [59]. In *H. pylori* infected mice, the relative abundance of *Lachnospiraceae* and *Odoribacter* decreased. However, after treating with *L. casei* T1, the relative abundance of *Lachnospiraceae* and *Odoribacter* not only increased, but also was higher than Group C. This indicated that treatment with *L. casei* T1 could improve the intestinal environment by promoting the growth of beneficial bacteria. Although the relative abundance of *Bifidobacterium* in Group H + T1 (0.091%) was significantly lower compared to Group C (2.99%), it was higher than that in Group H (0.030%). The elevated relative abundance of *Helicobacter* in Group H + T1 might be due to *L. casei* T1 inhibiting the colonization of *H. pylori* in the stomach, resulting in a large amount of *H. pylori* entering the intestine.

## Discussion

It is widely recognized that diet affects the incidence of many diseases [60]. For instance, bearberry and cranberry juice were reported to treat urinary tract infections [61]. Foods that have sufficient effects to improve health or reduce disease are called functional foods. Functional foods, including probiotics, have become an established dietary trend. Probiotic foods not only preserve probiotic viability during product manufacture and shelf life, but also exert their beneficial effect in consumers’ gastrointestinal tract [62]. The bacteriostasis of probiotics is by producing acidic substances, inhibiting bacterial adherence, or producing antibacterial substances [63]. Enhancement of mucosal barrier could be another important pathway that probiotics confer benefits to the host [64]. Studies have shown that probiotics have a variety of health benefits, such as anti-obesity, anti-inflammatory, and immune-boosting [65]. It has been shown the antimicrobial effect of *Lactobacillus* species on *H. pylori* infection in vivo, achieved by the unleash of bacteriocins and the ability to decrease adherence of *H. pylori* to epithelial cells [66]. Our previous studies proved *L. casei* T1 possessed excellent acid tolerance and a high survival rate in the stomach [26, 27]. *L. casei* T1 also could suppress adhesion of *H. pylori* strains in vitro [28]. As a result, *L. casei* T1 helps to competitively inhibit the colonization and absorption of nutrients of *H. pylori*. Probiotics also can inhibit or kill *H. pylori* by producing substances in the stomach [67]. Earlier studies showed that bacteriocins secreted by *L. casei* T1 had broad-spectrum antimicrobial activities. In this study, we demonstrated *L. casei* T1*’*s fermentation broth containing bacteriocin and organic acid can effectively inhibit *H. pylori* proliferation and had a better bacteriostatic effect than bacteriocin only (Table 1) [26]. It is evident from Figs. 1 and 2 that *L. casei* T1 significantly attenuated the expression of inflammatory factors induced by *H. pylori *in vitro*. This* confirmed that *L. casei* T1 can attenuate or even suppress harmful inflammatory responses during *H. pylori* infection in vitro*.* We also constructed *H. pylori-*infected murine models, which were verified to be successful by rapid urease experiment and agarose gel electrophoresis. The capacity to improve infection symptoms of *H. pylori* was finally confirmed in vivo.

Recent findings have suggested that probiotics performed systemic anti-inflammatory effects [68, 69]. Our results of blood routine are consistent with it (Table 2). Group H and Group H + T1 had a much higher level of leukocytes and neutrophils than Group C, indicating successful modeling of *H. pylori* infection. *H. pylori* can be colonized in the stomach through the production of urease, which protects it from the acidic environment by converting urea into bicarbonate and ammonia [70, 71]. So, the deeper the color of rapid urease test, the more serious *H. pylori* infection. The test paper color of Group H was significantly redder than Group H + T1 (Fig. 4), showing *L. casei* T1 treatment could substantially improve the prognosis for *H. pylori* infection and inhibit colonization rate in vivo. Compared to Group H, the mRNA expression of *MUC5AC* of Group H + T1 was markedly reduced (Fig. 5), also illustrating *L. casei* T1 treatment could help the body struggle with *H. pylori* colonization. Excessive inflammation causes tissue damage and chronic disease, including autoinflammatory and cardiovascular diseases [72–74]. Studies have revealed that TNF-α was over-expressed in patients with *H. pylori* infection [75]. The *TNF-α* mRNA expression of Group H + T1 was markedly lower than Group H (Fig. 5, *P* < 0.01). Some comparative studies showed that *H. pylori* infection may promote the expression of CRP [76, 77]. The results showed that *L. casei* T1 could alleviate the high CRP level by *H. pylori* infection (Fig. 6A). HE staining was used for observing mucosal inflammation and tissue damage [78]. After 4 weeks of *L. casei* T1 treatment, HE staining (Fig. 7) showed Group H + T1 exhibited lighter inflammation than Group H. The immunohistochemistry results (Fig. 8) also indicated that *L. casei* T1 treatment inhibited the overexpression of NF-κB protein triggered by *H. pylori* infection. The results of 16S sequencing showed that gut microbiome diversity and composition after treating *H. pylori* with *L. casei* T1 were closer to the control group. These results showed that *L. casei* T1 treatment was effective in mitigating the inflammatory response caused by *H. pylori*. As known, oxidative stress and inflammation are mutually dependent and connected [79, 80]. The change of ROS also can reflect inflammatory conditions of the body [81]. Oxidative stress is a harmful process that can negatively affect several cellular structures, such as membranes, lipids, proteins, lipoproteins and DNA [82, 83]. Studies show that *H. pylori* infection increases oxidative stress in vivo [84–86]. Reducing oxidative stress is beneficial for preventing the development of *H. pylori*-associated gastric diseases [87]. We have tested the indicators of oxidative stress. Despite the level of MDA shows that both Group H and H + T1 produce oxidative stress, but the MDA concentration of Group H + T1 was significantly lower than Group H (*p* < 0.001). At the same time, GSH-PX and SOD which play as an antioxidant of Group H + T1 was more than Group H. These findings clearly indicated that *L. casei* T1 was able to attenuate *H. pylori*-induced ROS production in vivo*.*

## Conclusions

In this paper, we revealed that *L. casei* T1 could alleviate *H. pylori*-induced inflammation and oxidative stress in vivo and in vitro*.* Although taking *L. casei* T1 cannot completely eradicate *H. pylori*, it can effectively reduce the damage caused by *H. pylori* infection. Therefore, further research could explore thoroughly eradicate *H. pylori* by *L. casei* T1 in conjunction with antibiotic and reveal the differences in prevention and treatment of *H. pylori*-induced gastric mucosal inflammation.

## Data Availability

The dataset supporting the conclusions of this article are available in the National Center for Biotechnology Information (NCBI) Sequence Read Archive (SRA) repository, BioProject ID PRJNA857345 (http://www.ncbi.nlm.nih.gov/bioproject/857345). The data will be released to the public when the manuscript is formally accepted for publication.
